# Ipecacuanha in Large Non Emetic Dose in the Treatment of Cholera

**Published:** 1884-09-20

**Authors:** 


### IPECACUANHA IN LARGE NON-EMETIC DOSE IN THE TREATMENT OF CHOLERA.

Among the numerous plans of treatment that have been recommended or actually adopted in the treatment of true, or Asiatic cholera, there is none that, from past experience, seems more deserving of a faithful extended trial than that by ipecacuanha, as presented to the profession with such force and ability by Surgeon A. A. Woodhull, U. S. A.* The method of treatment of dysentery which was advocated by the author, by "large non-emetic doses," has become widely known and is generally practised, but the fact seems not to have been properly appreciated that he also insisted upon the possible value of this method of treatment in Asiatic cholera, the full title of his essay being "Clinical Studies with Large Non-Emetic Doses of Ipecacuanha, with a Contribution to the Therapeusis of Cholera." Without attempting an analysis of this valuable paper, we merely repeat the caution of the author, who especially enjoins upon those who adopt the treatment in their practice to adhere to the non-emetic method. Large doses, from a scruple to a drachm of the powdered root, may be given without disturbing the stomach, if the patient remain quietly in the recumbent posture after its administration and abstain for a time from drinking fluids. Very often, however, it is necessary to insure the desired result by the preliminary administration of a full dose of laudanum. The strikingly favorable results thus obtained by the administration of ipecacuanha in dysentery, in hemorrhage and in collapse would certainly warrant the further extension of this treatment to analogous conditions occurring in the course of cholera. Nor is the recommendation for its employment in cholera without support from clinical experience. M. Bourdon, in tbe wards of La Charite, has used it with decided success in the treatment of cholera infantum, which so closely re-

* In a report to the Surgeon-General in October, 1874, which afterwards appeared in full in the Atlanta Medical and Surgical Journal.

sembles the true cholera.† In fact, it was reported by M. Decugis (mentioned by Woodhull) that at the first appearance of the cholera in 1832, the physicians, struck by its resemblance to dysentery, proposed ipecac, and it was even considered a specific for cholera by Grisolle; but further experience taught that, when used in the ordinary way, it was of slight utility, and this opinion is generally echoed by our text-books.

The treatment of Dr. Woodhull is, on the contrary, based upon the effects of ipecacuanha when administered in a very different manner, in large, non-emetic dose, in which it acts as a sedative, as pointed out by Waring, who also recommended its use in cholera.—*Medical Times.*

† Reported by Chouppe in Recherches therapeutiques et physiologiques sur l'Ipeca, Paris, 1874.

